# Health Research and the World Humanitarian Summit—Not a Thousand Miles Apart

**DOI:** 10.1371/journal.pmed.1002027

**Published:** 2016-05-23

**Authors:** The PLOS Medicine Editors

**Affiliations:** The *PLOS Medicine* Editors are Clare Garvey, Thomas McBride, Linda Nevin, Larry Peiperl, Amy Ross, Paul Simpson, and Richard Turner.

## Abstract

As the World Humanitarian Summit and the World Health Assembly convene on the same day in different cities, The *PLOS Medicine* Editors support a role for health in the UN Agenda for Humanity.

May 23–24, 2016, marks the first World Humanitarian Summit (WHS), convened in Istanbul by United Nations Secretary-General Ban Ki-Moon following three years of preparatory consultations [1]. In an effort to resolve current humanitarian crises and avert future ones, the event aims to bring together heads of state, leaders of crisis-affected communities, representatives from industry, multilateral and nongovernment organizations, and other groups involved in humanitarian crises. Remarkably, the same dates bring a key meeting for another UN agency: the 69th World Health Assembly, through which member states govern the World Health Organization (WHO), meets May 23–28 in Geneva, more than 1,000 miles away.

Health crises are both cause and consequence of humanitarian crises. As part of the WHS consultation process, WHO has emphasized that “[t]he health and well-being of affected populations is the ultimate goal of humanitarian action” [2]. The Secretary-General’s report for the WHS, which includes the five-section Agenda for Humanity, describes the present situation in cataclysmic terms that include global threats to health:

Brutal and seemingly intractable conflicts have devastated the lives of millions of people…. More countries are slipping into fragility, marked by extreme poverty…. Violent extremism, terrorism and transnational crime are creating persistent instability. Growing economic inequality within countries and the widening gap between the rich and the poor are further marginalizing the most vulnerable people in society. Climate change continues to cause increased humanitarian stress as it exacerbates food insecurity, water scarcity, conflict, migration and other trends. Disasters are becoming more frequent and intense. Pandemics, epidemics and other global health threats continue to emerge frequently, and at worrying levels… [3]

In the context of this clear common ground, it seems ironic that a scheduling conflict effectively precludes health ministers and WHO leadership from attending both WHA and WHS. As journal editors, we are prompted to ask in turn how the core concerns of our work—medicine and health research—align with the priorities of the Agenda for Humanity.

Medical interventions appear in the Secretary-General’s report as part of Core Responsibility 2 (“uphold the norms that safeguard humanity”), in which the human rights of civilians are noted to include access to humanitarian medical services, care for the sick and wounded, and protection against attacks on hospitals and medical workers. We support this identification of medical services as crucially important. Having joined the call in 2014 for an end to deliberate attacks on medical services in conflict settings [4], we can scarcely conceive language sufficient to condemn the repeated bombing of hospitals in air-strikes that have since become widespread [5–7]. Core Responsibility 3 (“leave no one behind”) includes recognition that women and girls in crisis settings, as well as displaced people with disabilities and older people, are particularly at risk of poor access to health programs. Even in a document clearly intended to emphasize critical needs for political leadership and financing, these few mentions of health are notable for their brevity.

Limited mention of health and medicine may reflect the prominence of UN Sustainable Development Goal 3, devoted specifically to health and well-being; to expect each initiative to cover every important topic would invite loss of focus. Even so, moving from medical services to medical research, one finds the word “research” only once in the report’s 62 pages of text; the words “science” and “scientific” do not appear at all. Rather, the report emphasizes the need to collect, share, and analyze data for the purpose of assessing need, anticipating crises, and monitoring responses: “All actors should commit to consolidating available data in open and widely accessible databases, with adequate security and privacy protection…to inform joint analysis and a common picture of the most pressing risks. This common picture should be used to set ambitious targets towards implementing and financing preparedness and risk management strategies” [3].

In many crisis situations, a surveillance and monitoring approach may be more appropriate than the classic scientific approach of designing studies to test hypotheses and generate new, generalizable knowledge. At other times, the two approaches may overlap, testing the limits of the scientific method, as we cannot expect data obtained in a crisis situation to attain the methodological rigor of prospectively designed studies in controlled settings. In addition to publishing scientific studies, a journal may raise awareness of issues and thoughtful approaches to solutions. (Recent examples in this journal include essays on the role of physicians in refugee detention centers in Australia [8] and statistical approaches to making the most of limited mortality data on forced migrants, such as those in Southern Sudan and Iraq [9].) Nonetheless, we believe that a scientific approach has more to offer than may be apparent from the wording of the Secretary-General’s report. While opportunities to obtain sound data in crises may be fleeting and perilous, and opportunities to replicate conclusions uncertain, the results can still be illuminating and useful.

One of *PLOS Medicine’s* more frequently viewed and cited articles attributed one-third of deaths following the United States invasion of Iraq not to direct violence but to indirect causes such as failures of health, sanitation, and other systems [10]. We believe that this research, based on household survey reports, analyzed against a historical comparison group, and unlikely to be replicated directly, has substantially advanced understanding of the scope of humanitarian crises that follow war. While the results are necessarily estimates, had the investigation not been conducted using established scientific methodology, the reliability of the conclusions would be impossible to assess.

The widespread occurrence of current atrocities and the threat of future devastation demand action. We applaud the potential of WHS to align the necessary agencies and resources. Yet, the very need for such a summit suggests that, despite advances in human rights over recent decades, governments and agencies with the capacity to act still lack evidence for prioritizing and pursuing effective action. In humanitarian crises, collecting and monitoring data will be necessary but not sufficient; a sustainable impact on effects requires an understanding of causes, and responsible action requires investigation of how best to implement what is understood [11].

While science cannot by itself resolve disasters of such profound political and ethical dimensions, research can bring objectivity and innovation to understanding the causes of humanitarian crises and evaluating approaches to their prevention and resolution. We hope that discussions sparked by the Istanbul Summit will more closely integrate health objectives and clarify the role that original research should play in the global response to humanitarian crises.

## Abbreviations

WHO: World Health Organization
WHS: World Humanitarian Summit
